# Predicting recurrence and metastasis risk of endometrial carcinoma *via* prognostic signatures identified from multi-omics data

**DOI:** 10.3389/fonc.2022.982452

**Published:** 2022-08-19

**Authors:** Ling Li, Wenjing Qiu, Liang Lin, Jinyang Liu, Xiaoli Shi, Yi Shi

**Affiliations:** ^1^ Department of Gynecological Oncology Surgery, Fujian Cancer Hospital, Fujian Medical University Cancer Hospital, Fuzhou, China; ^2^ Science System Department, Geneis Beijing Co., Ltd., Beijing, China; ^3^ Qingdao Geneis Institute of Big Data Mining and Precision Medicine, Qingdao, China; ^4^ Department of Molecular Pathology, Fujian Cancer Hospital, Fujian Medical University Cancer Hospital, Fuzhou, China

**Keywords:** endometrial carcinoma, recurrence and metastasis, lncRNA, CNV, mRNA, miRNA, prediction model

## Abstract

**Objectives:**

Endometrial carcinoma (EC) is one of the three major gynecological malignancies, in which 15% - 20% patients will have recurrence and metastasis. Though there are many studies on the prognosis on this cancer, the performances of existing models evaluating the risk of its recurrence and metastasis are yet to be improved. In addition, a comprehensive multi-omics analyses on the prognostic signatures of EC are on demand. In this study, we aimed to construct a relatively stable and reliable model for predicting recurrence and metastasis of EC. This will help determine the risk level of patients and choose appropriate adjuvant therapy, thereby avoiding improper treatment, and improving the prognosis of patients.

**Methods:**

The mRNA, microRNA (miRNA), long non-coding RNA (lncRNA), copy number variation (CNV) data and clinical information of patients with EC were downloaded from The Cancer Genome Atlas (TCGA). Differential expression analyses were performed between the recurrence or metastasis group and the non-recurrence/metastasis group. Then, we screened potential prognostic markers from the four kinds of omics data respectively and established prediction models using three classifiers.

**Results:**

We achieved differential expressed mRNAs, lncRNAs, miRNAs and CNVs between the two groups. According to feature selection scores by the random forest algorithm, 275 CNV features, 50 lncRNA features, 150 miRNA features and 150 mRNA features were selected, respectively. And the prediction model constructed by the features of lncRNA data using random forest method showed the best performance, with an area under the curve of 0.763, and an accuracy of 0.819 under 10-fold cross-validation.

**Conclusion:**

We developed a computational model using omics information, which is able to predicting recurrence and metastasis risk of EC accurately.

## Introduction

Endometrial carcinoma (EC) is a kind of epithelial malignant tumor occurring in the endometrium and is one of the three major gynecological malignancies (1–3). In North America and Europe, it is the fourth leading cancer following breast cancer, lung cancer, colorectal tumor in terms of incidence (4). In China, the incidence of the disease is also increasing year by year and is second only to cervical cancer (5). Obesity, hormonal and metabolic disorders are particularly closely related to the occurrence of EC (6). Its clinical treatment is mainly surgical resection, supplemented by radiotherapy and drug treatment. Although most patients are at the early stage when diagnosed and have a good prognosis, 15% - 20% of patients will have recurrence and metastasis (7–9). The presence of poor prognosis of recurrence or metastasis is the main cause leading to the death of EC patients (10, 11). Therefore, accurate prediction of the recurrence and metastasis of endometrial cancer as early as possible and performed targeted adjuvant therapy are essential to improve the survival rate of EC patients. In fact, it is difficult to identify patients with a high risk of recurrence and metastasis in the early stage. Traditionally, clinicians usually predict the risk of recurrence and metastasis by pathological type, histological grade, depth of myometrial invasion, lymphatic metastasis and extrauterine lesions, and monitor the development of the disease through patients’ regular radiologic examination and laboratory examinations (12–15).

Nowadays, with the development of liquid biopsy technology and the popularization of artificial intelligence in the field of medical images, there are many new explorations and novel methods in predicting tumor recurrence and metastasis (16–22). For example, Wu et al. developed a deep convolutional neural network (CNN) model to predict the risk of recurrence and metastasis from hematoxylin and eosin (H&E) stained sections of lung cancer (23). For estimating the risk of recurrence and metastasis in patients with HER2-positive breast cancer, Yang et al. constructed a novel multimodal fusion model integrating H&E images and clinical characteristics (20), with an area under the curve (AUC) of 0.72 in the independent testing data. Feng et al. identified that detection of somatic mutations of ctDNA could predict recurrence of EC effectively and stably (16). Ye et al. developed a deep convolution network to predict cervical cancer metastasis and recurrence risk (24). Based on the study results of The Cancer Genome Atlas (TCGA), endometrial cancer was classified into four categories according to the mutation spectrum, somatic copy number alterations (SCNAs) and microsatellite instability (MSI): DNA polymerase epsilon (POLE) ultramutated, high microsatellite instability (MSI-H), copy-number low, and copy-number high (25). TCGA molecular typing has initially shown good application prospects in predicting the prognosis of endometrial cancer patients and has been listed in the national comprehensive cancer network (NCCN), which may affect post-surgical adjuvant treatment. However, no applicable prognostic prediction models only based on genomics have been found by retrieving concerned literatures (8, 10).

In this study, all sequencing data and clinical information of patients with EC from TCGA (http://cancergenome.nih.gov/) were downloaded and organized to study the association between gene mutation/expression and recurrence or metastasis of EC. Specifically, we first compared the differential expressions of mRNA, long non-coding RNA (lncRNA) and microRNA (miRNA) between patients with recurrence or metastasis and patients without recurrence or metastasis using the DESeq2 package, and then analyzed differences of copy number variations (CNVs) between the two groups by rank-sum test. Furthermore, we analyzed the function of these differential genes, and discussed the molecular mechanism of recurrence and metastasis of EC. After that, characteristic variables were selected by a random forest (RF) algorithm with feature selection and were used to establish prognostic prediction models using three different classifiers. Finally, the RF model based on lncRNA showed the best performance among the twelve models.

## Materials and methods

### Study participants

TCGA is a great cancer genome project which has produced genomic, epigenomic, transcriptomic and proteomic data of more than 20,000 cancer patients covering multiple cancer types. These data can help researchers have a more comprehensive understanding of cancer and improve the level of cancer screening, diagnosis and treatment. Clinical information of 548 patients with EC was downloaded from the TCGA data portal, including 204 patients without recurrence or metastasis, 43 patients with recurrence or metastasis and 301 patients without information of recurrence and metastasis. In addition, mRNA sequencing data of 543 EC patients, miRNA data of 538 EC patients, lncRNA data of 537 EC patients and CNV data of 534 EC patients were downloaded. Then, we matched the data according to the patient ID, and selected patients with complete omics data and prognostic information into the study.

### Difference analysis

The expression data of mRNAs, lncRNAs and miRNAs were displayed as reads per million (RPM) and the expression levels were normalized by DESeq2 (26) package of R language for difference analysis. Then the differentially expressed mRNAs, lncRNAs and miRNAs were calculated by DESeq2 with Padj < 0.05 and the absolute log2FC > 1 as the cutoff value, respectively. The CNVs of two groups (recurrence and metastasis group and non-recurrence/metastasis group) were analyzed by SPSS statistical software and significant differences were screened by rank-sum test with P < 0.005 as the threshold.

To explore the potential biological functions of these differential genes and the signal pathways they may participate in, we performed Gene Ontology (GO) (27, 28) and Kyoto Encyclopedia of Genes and Genomes (KEGG) enrichment analyses by employing clusterProfiler R package (29) with *p_*value < 0.05 and *q_*value > 1 as the threshold.

### Feature selection for modeling

The patients in the recurrence and metastasis group and in the group without recurrence or metastasis were divided into the training set and the test set with the ratio of 7:3, respectively. After the division, the patients were fixed in the training set or the test set, that is, in different omics analyses, the same patient was always in the training set or test set.

For each omics data of the training set, a feature selection algorithm based on RF was applied to screen important features (30–33). Specifically, we screened the characteristic variables with scores according to Gini index. Then the features were grouped in steps of 25 and performed 10 fold cross-validation and scored to confirm the final number of features.

### Model construction and comparison

Based on the selected characteristic parameters, RF, logistic regression and support vector machine classifiers were chosen for model construction to select the best model to predict the recurrence or metastasis of patients with endometrial cancer. Specifically, omics data in the training set were grid searched in each classifier to select the best super parameter, and then the final super parameter was determined through 10 fold cross-validation. Finally, we obtained 12 prediction models and compared the prediction performance mainly using the AUC of receiver operating characteristic (ROC) curves, precision and accuracy.

## Results

### A brief study design of exploring molecular mechanism and establishing prediction model

**Figure 1:**
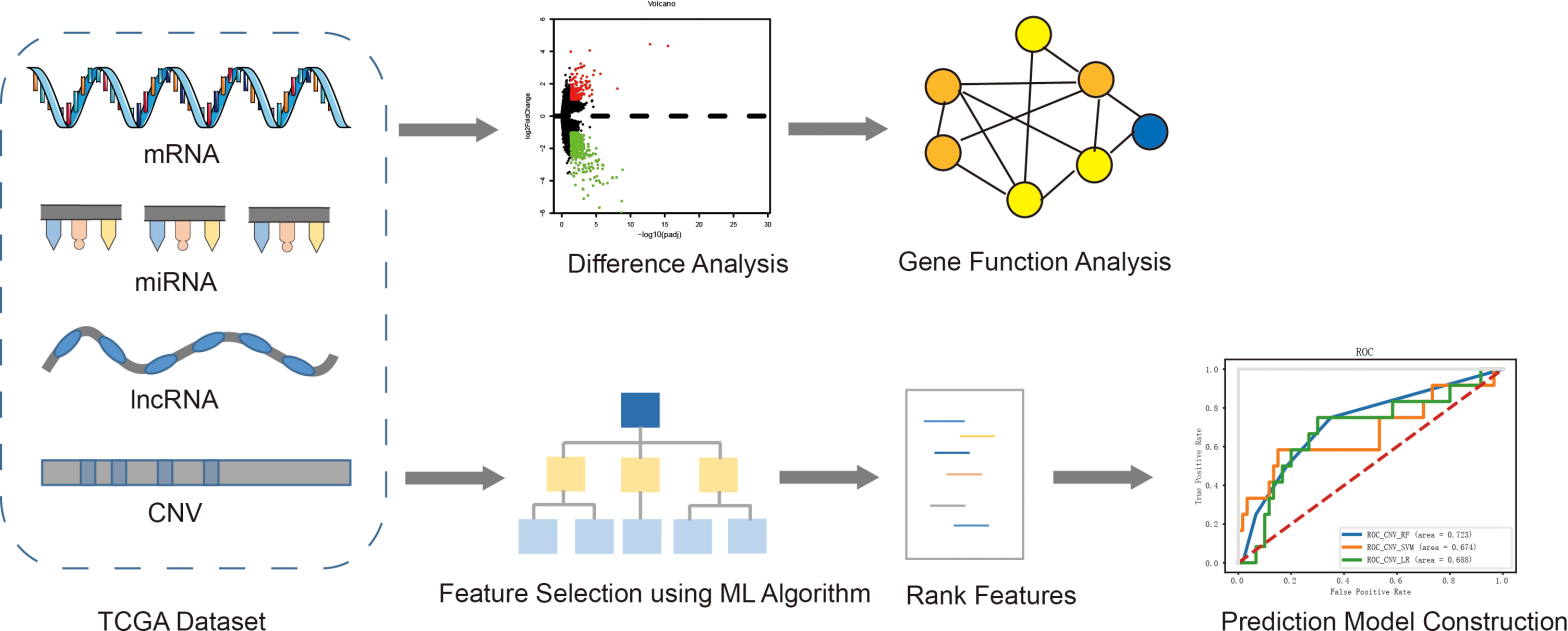
The overall pipeline of this study, including the following main steps: 1) Obtained four kinds of omics data (mRNA, miRNA, lncRNA and CNV) and clinical information of EC patients from TCGA; 2) Difference analysis between two groups and function analysis of differential genes; 3) Feature selection and construction of prediction model.+.

The overall process of exploring the molecular mechanism of recurrence and metastasis, and establishing risk prediction models using a machine learning algorithm was described in **Figure 1**. Firstly, four kinds of omics data and clinical information of EC patients were downloaded from TCGA, and then the patients were divided into two groups according to the prognosis status. Secondly, differential expression analysis was performed between the recurrent and metastatic group and non-recurrent metastatic group. Furthermore, we analyzed the function of differential genes using GO and KEGG. At the same time, characteristic variables were selected by a RF algorithm with feature selection and were used to establish prognostic prediction models using three different classifiers.

### Clinicopathological features of patients with EC

After matching data according to the patient ID, 238 EC patients with both prognostic information and four kinds of omics data were obtained. Of these patients, 39 patients (16.39%) had cancer recurrence or metastasis whereas 199 patients (83.61%) had no recurrence or metastasis. Clinical and pathological information of these two groups of patients in this study was shown in **Table 1**. Because some information is absent, such as lymph node, progesterone receptor and estrogen receptor status, only some factors that may be related to the prognosis of patients (7, 34) were selected for statistical analysis. As can be seen, clinical stage was significantly associated with recurrence or metastasis in this set of data, with a *P_*value of 0.000656. Whereas, there were no significant differences between the recurrent or metastatic group and non-recurrent or metastatic group in ages (median), body mass index (BMI) and pathological type.

**Table 1:** Summary of clinical information of patients with EC.

Clinicopathologic variable	Category	Recurrence or metastasis	Without recurrence or metastasis	*P_*value
Age (years)	Median	62	61	1.000
Clinical stage	I	18	124	0.000656
	II	5	28	
	III	11	45	
	IV	5	2	
Pathological type	Endometrioid carcinoma	23	142	0.179
	Non-endometrioid carcinoma	16	57	
BMI	≤28	7	62	0.845
	>28	32	137	

### Results of differential expression analysis

**Figure 2:**
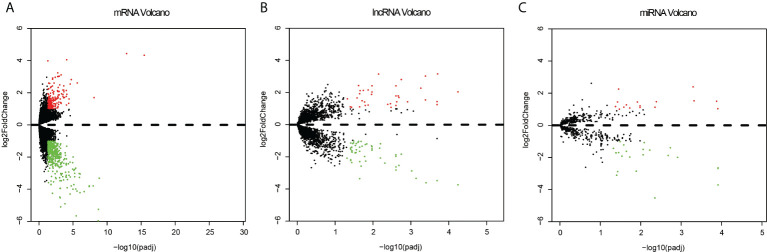
Volcano plots of the differentially expressed mRNAs **(A)**, lncRNAs **(B)** and miRNA **(C)** between the recurrent or metastatic group and non-recurrent or metastatic group. Red represents up expression and green represents down expression. Black indicates the expression with both the absolute log2FC > 1 and Padj < 0.05. The X axis shows an adjusted P value and the Y axis shows a log2FC.

Among the expression data of 17,958 mRNAs, 592 mRNA genes were expressed significantly different between the recurrent and metastatic group and non-recurrent or metastatic group, with 169 up-regulated genes and 423 down-regulated genes in the recurrent or metastatic group compared to the non-recurrent or metastatic group (**Figure 2A**). And 3,352 differentially lncRNAs were achieved, in which 87 lncRNAs were significantly different, with 51 down-regulated and 36 up-regulated (**Figure 2B**). In addition, there were 687 differentially expressed miRNAs, in which 39 miRNAs were significantly different, with 23 down-regulated and 16 up-regulated (**Figure 2C**). Heatmaps of the top 50 differentially expressed mRNA, lncRNA and miRNA were shown in **Supplementary Figures 1**–**3**. As CNVs had been reported to affect the recurrence of EC (34), it was selected separately for analysis. Finally, 939 significantly different CNVs were got after analyzing by SPSS statistical software with P < 0.005 as the threshold.

**Figure 3:**
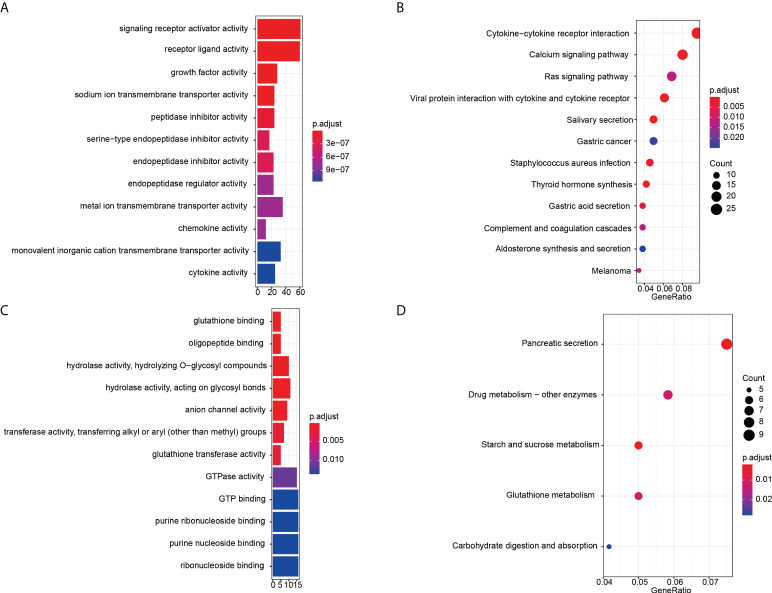
Enrichment analysis results. GO enrichment analysis results of significantly differentially expressed mRNAs **(A)** and CNVs **(C)**. The x-axis is gene counts, the y-axis is GO terms of molecular function. KEGG pathways of the differentially expressed mRNAs **(B)** and CNVs **(D)**. The x-axis is the ratio of genes in the corresponding pathway, and the y-axis is the name of the pathway.

Then GO and KEGG enrichment analyses were performed to explore the function and involved signal pathways for further investigating the prognostic value and molecular mechanisms. For significantly differentially expressed mRNAs, the top twelve molecular functions with the highest proportion of genes were displayed in **Figure 3A**, and first twelve enriched signaling pathways were shown in **Figure 3B**. We found that the molecular functions of these differentially expressed mRNAs mainly enriched in signaling receptor activator activity, receptor ligand activity, growth factor activity, sodium ion transmembrane transporter activity, peptidase inhibitor activity, serine-type endopeptidase inhibitor activity, and so on. And the results of KEGG pathway analysis indicated that the recurrence or metastasis of EC may be correlated to the regulation of cytokine-cytokine receptor interaction, Calcium signaling pathway, Ras signaling pathway, viral protein interaction with cytokine and cytokine receptor. GO enrichment analysis results and KEGG pathways of the different CNVs were displayed in **Figures 3C, D**, respectively. From the perspective of molecular function, these mainly focus on glutathione binding, oligopeptide binding, hydrolase activity, hydrolyzing O-glycosyl compounds, hydrolase activity, acting on glycosyl bonds, anion channel activity, transferase activity, transferring alkyl or aryl (other than methyl) groups. The genes with significant CNV differences are mainly involved in the following five pathways: pancreatic secretion, drug metabolism - other enzymes, starch and sucrose metabolism, glutathione metabolism and carbohydrate digestion and absorption.

### Modeling using lncRNA showed the best prediction performance

**Figure 4:**
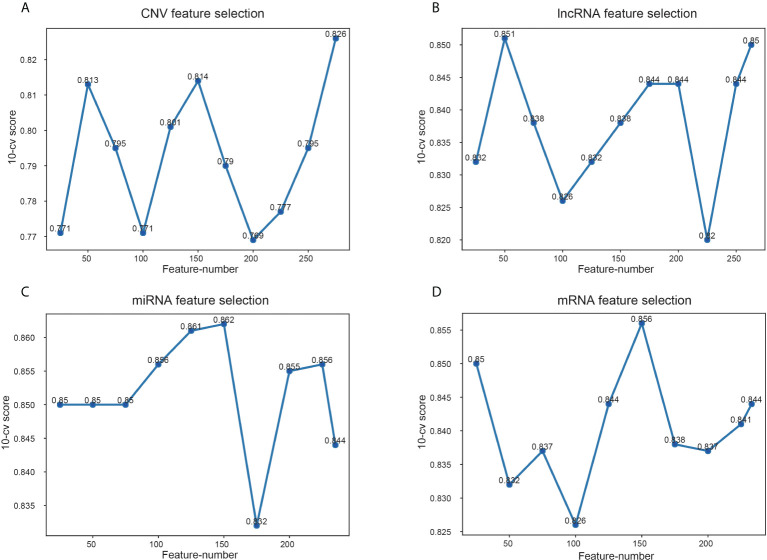
Scores of different feature number selection based on omics data. **(A)** Feature selection of CNV, **(B)** feature selection of lncRNA, **(C)** feature selection of miRNA, **(D)** feature selection of mRNA. The x-axis is feature numbers, the y-axis is the 10-CV score.

Among the data of EC downloaded from TCGA, there are 17958 mRNA expression information, 7315 lncRNA expression information, 1881 miRNA expression information and 16383 copy number variation information. Variable selection for these biological data was performed using the RF to determine variable importance measures. The scoring of different number of features screened by RF is shown in **Figure 4**. The features with the highest 10-CV score were selected for model construction. Specifically, 275 features were chosen from CNV data because the score of 275 features was 0.826, significantly higher than other feature combinations (**Figure 4A**). And 50 lncRNA features were selected as the score was 0.851, which was higher than others (**Figure 4B**). For the other two kinds of genomic data, 150 features of miRNA and mRNA were selected, with the highest score of 0.862 and 0.856, respectively (**Figures 4C, D**).

**Figure 5:**
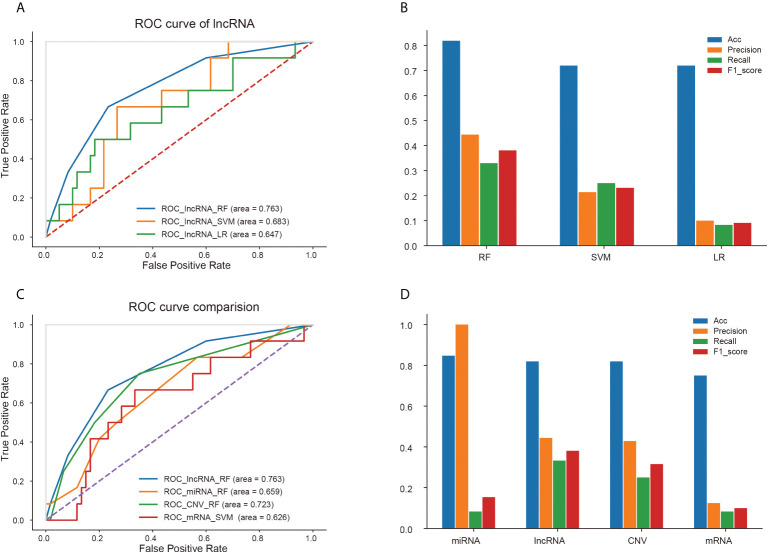
Prediction performance of different models based on four kinds of omics data. **(A)** ROC curves of three models based on lncRNA data. **(B)** Accuracy, precision, recall and F1-score of three models for lncRNA signatures. Comparison of ROC curves **(C)** and four properties **(D)** of optimal models based on four kinds of omics data.

For each kind of omics data, three classifiers (RF, LR and SVM) were used to construct the prediction model. The ROC curves of three models based on lncRNA data were displayed in **Figure 5A**, because the model based on the characteristics of lncRNA data represented the best prediction performance. And accuracy, precision, recall and F1-score of the three models were shown in **Figure 5B**. The RF model constructed by the features of lncRNA data was able to predict recurrence or metastasis of EC with an AUC of 0.763, an accuracy of 0.819. The ROC curves of other models using omics variables were shown in **Supplementary Figures 4**. The ROC curves of models with the best prediction performance constructed by four omics data (lncRNA, mRNA, miRNA and CNV) were represented in **Figure 5C**, and the accuracy, precision, recall and F1-score of the prediction models were revealed in **Figure 5D**.

## Discussion

The Oncotype Dx (21 genes) and MammaPrint (70 genes) are two products for predicting recurrence and metastasis of breast cancer which have been internationally recognized (35). However, for patients with EC, there is no effective model based on molecular variations to evaluate the risk of recurrence and metastasis. EC is a malignant tumor, which often occurs in perimenopausal and postmenopausal women. It usually has a good prognosis if diagnosed early and treated appropriately. So, patients will benefit greatly when a product like Oncotype DX appears, that can help clinicians assess the recurrence risk of patients and adopt adjuvant treatment strategies according to different risk stratification. Previous studies have established prediction models based on clinical characteristics (14, 15) and combined clinical characteristics with molecular data (34). AUC value of the model using clinical features only was about 0.7, whereas M. D. Miller et al. used different kinds of molecular data, up to 5 categories, it may be difficult and expensive to apply clinically.

Here, starting from the data of TCGA, we analyzed the differences of mRNA expression, miRNA expression, lncRNA expression as well as CNVs between patients with recurrence and metastasis and non-recurrence or metastasis, and further analyzed their molecular biological functions and involved signal pathways, trying to explore the molecular biological mechanism of these differences and recurrence and metastasis. Although these molecules are related to recurrence and metastasis, it does not mean that these differential molecules can accurately and reliably predict recurrence and metastasis. To build the prediction model, it is still necessary to select the most appropriate feature combination by statistical methods (17, 34). Therefore, we used the feature selection algorithm of RF to filter features and selected different classifiers to establish models. Finally, the model using lncRNA data showed the best performance, with an AUC of 0.763, an accuracy of 0.819.

There are still several limitations in this study. Firstly, the sample size was limited and the survival time of tracking was not long enough, which may lead to inaccurate results. However, we have downloaded all samples information from TCGA, a relatively large-scale cancer genome database. Looking for more samples from other open databases or hospitals may be a solution. Secondly, the research on molecular mechanism was not deep enough (36–39). Maybe we should explore and discuss the mechanism of recurrence and metastasis of endometrial cancer in another study (40–42). Thirdly, a more advanced computational model can further improve the prediction accuracy as used elsewhere (43–46). Finally, this study lacked an independent validation set and did not develop a clinically scoring system and thresholds to discriminate risk of recurrence and metastasis. At present, we have not collected enough clinical samples for verification. We will continue to collect data to improve this study.

## Data availability statement

The original contributions presented in the study are included in the article/**Supplementary Material**. Further inquiries can be directed to the corresponding authors.

## Ethics statement

Ethical review and approval was not required for the study on human participants in accordance with the local legislation and institutional requirements. Written informed consent for participation was not required for this study in accordance with the national legislation and the institutional requirements.

## Author contributions

YS and XS designed the project. LLi, WQ, LLin, and JL collected and analyzed the data of patients with endometrial cancer. LLi and XS searched literatures and wrote the manuscript. All authors have approved the final version of the manuscript.

## Conflict of interest

WQ, JL, and XS were employed by the company Geneis Beijing Co., Ltd.

The remaining authors declare that the research was conducted in the absence of any commercial or financial relationships that could be construed as a potential conflict of interest.

## Publisher’s note

All claims expressed in this article are solely those of the authors and do not necessarily represent those of their affiliated organizations, or those of the publisher, the editors and the reviewers. Any product that may be evaluated in this article, or claim that may be made by its manufacturer, is not guaranteed or endorsed by the publisher.

## Supplementary material

Supplementary Figure 1 Heatmap of the top 50 differentially expressed mRNA.

Supplementary Figure 2 Heatmap of the top 50 differentially expressed lncRNA.

Supplementary Figure 3 Heatmap of the top 50 differentially expressed miRNA.

Supplementary Figure 4 Prediction performance of three kinds of models based on CNV data **(A)**, miRNA data **(B)** and mRNA data **(C)**.

## Abbreviations

EC, endometrial carcinoma; lncRNAs, long non-coding RNAs; CNV, copy number variation; NCCN, national comprehensive cancer network; TCGA, The Cancer Genome Atlas; AUC, area under the curve; ROC, receiver operating characteristic; RF, random forest; GO, Gene Ontology; KEGG, Kyoto Encyclopedia of Genes and Genomes.
