# Supplementary figures and images for: Predicting recurrence and metastasis risk of endometrial carcinoma via prognostic signatures identified from multi-omics data
Source: Front Oncol. 2022 Aug 19;12:982452. doi: 10.3389/fonc.2022.982452 (PMC9438970; doi:10.3389/fonc.2022.982452)

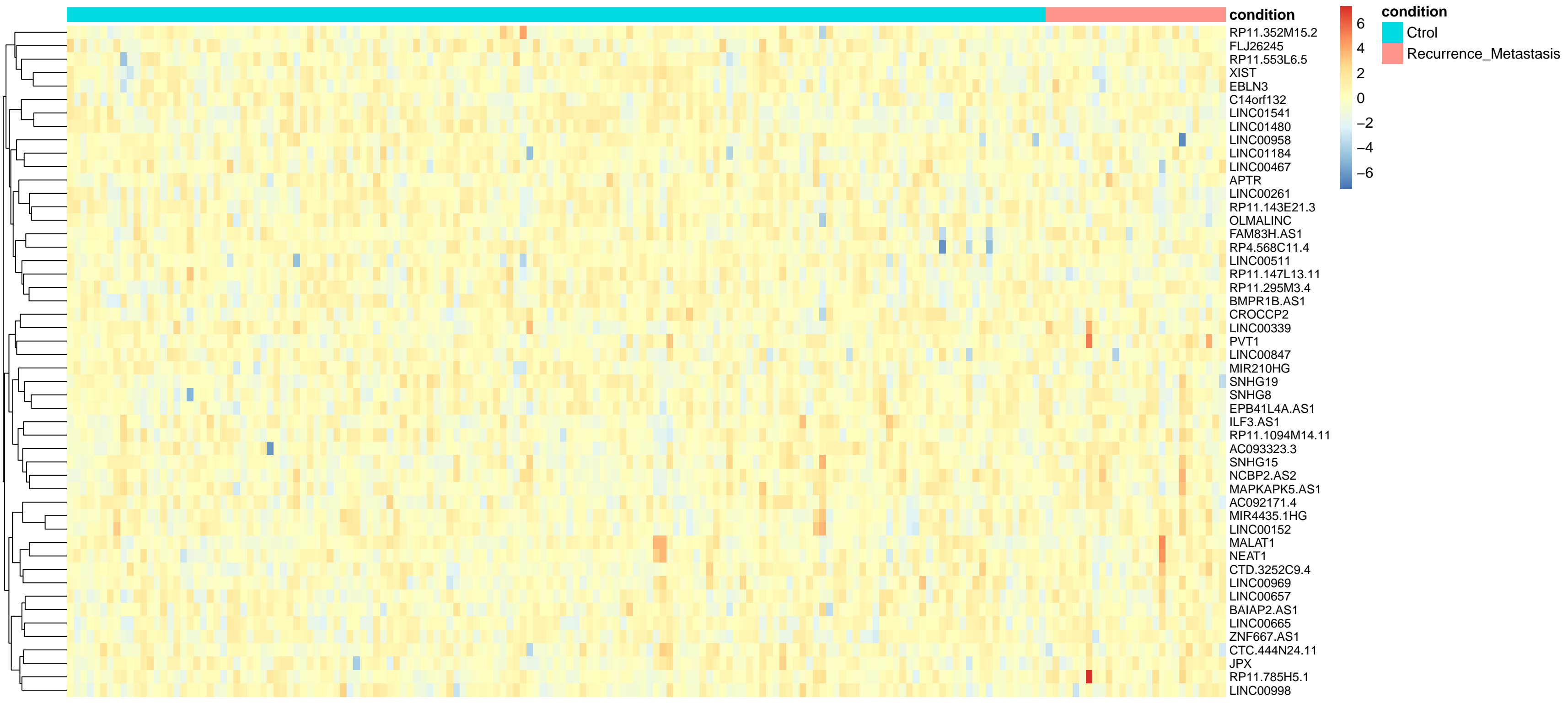

Supplement: Supplementary Figure 1 — Heatmap of the top 50 differentially expressed mRNA. [file DataSheet_1.pdf]

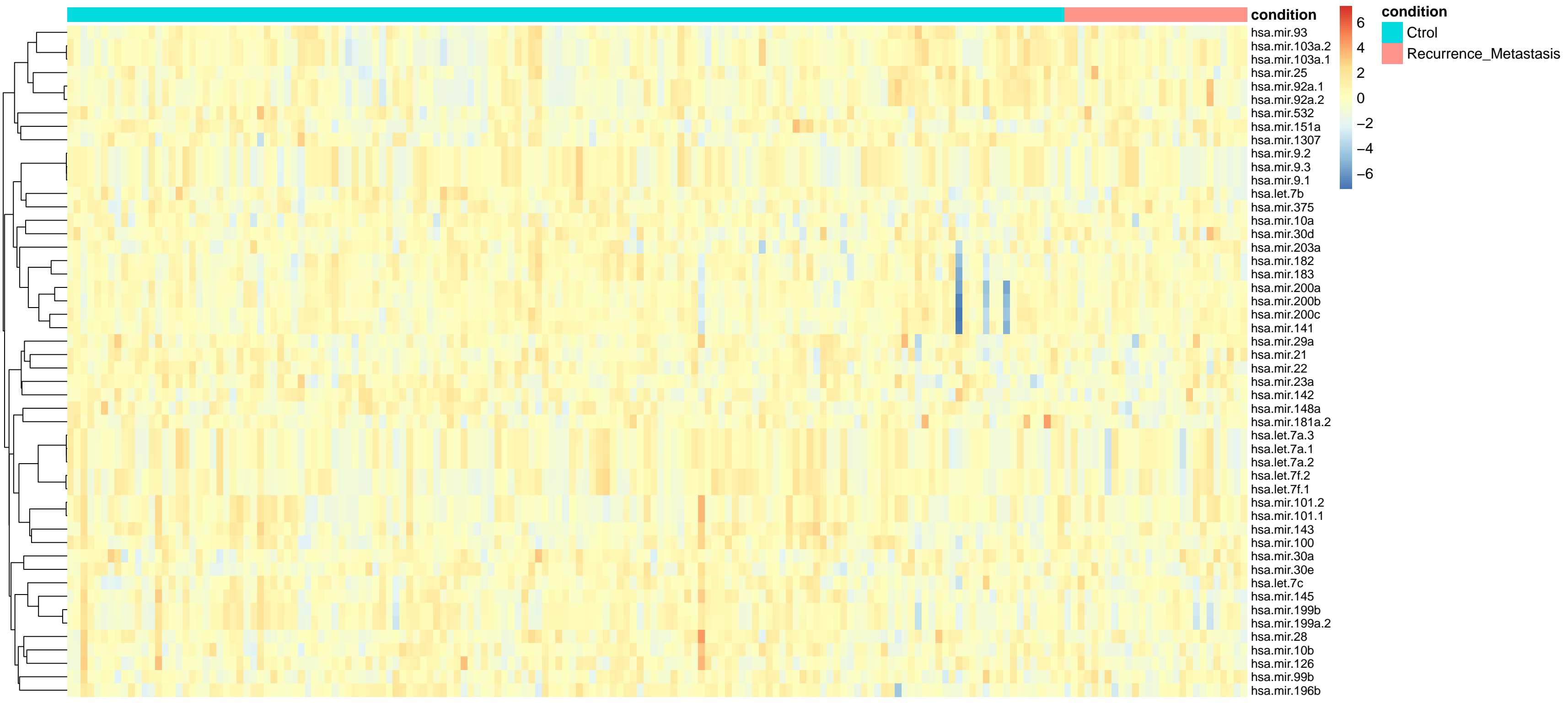

Supplement: Supplementary Figure 2 — Heatmap of the top 50 differentially expressed lncRNA. [file DataSheet_2.pdf]

A

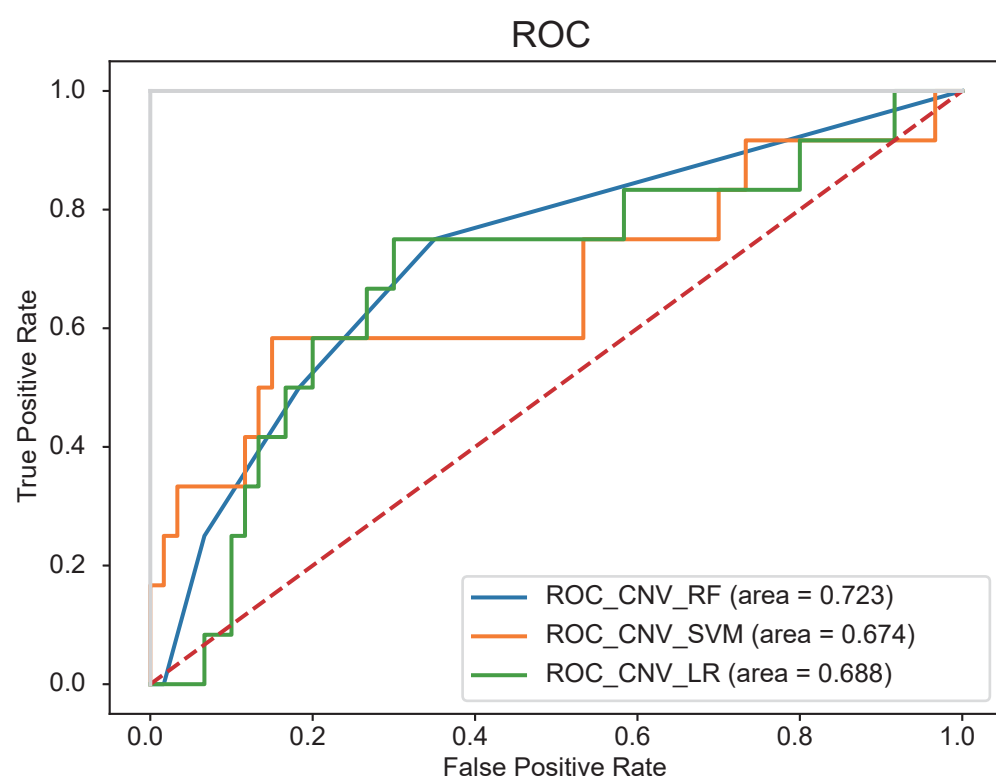

B

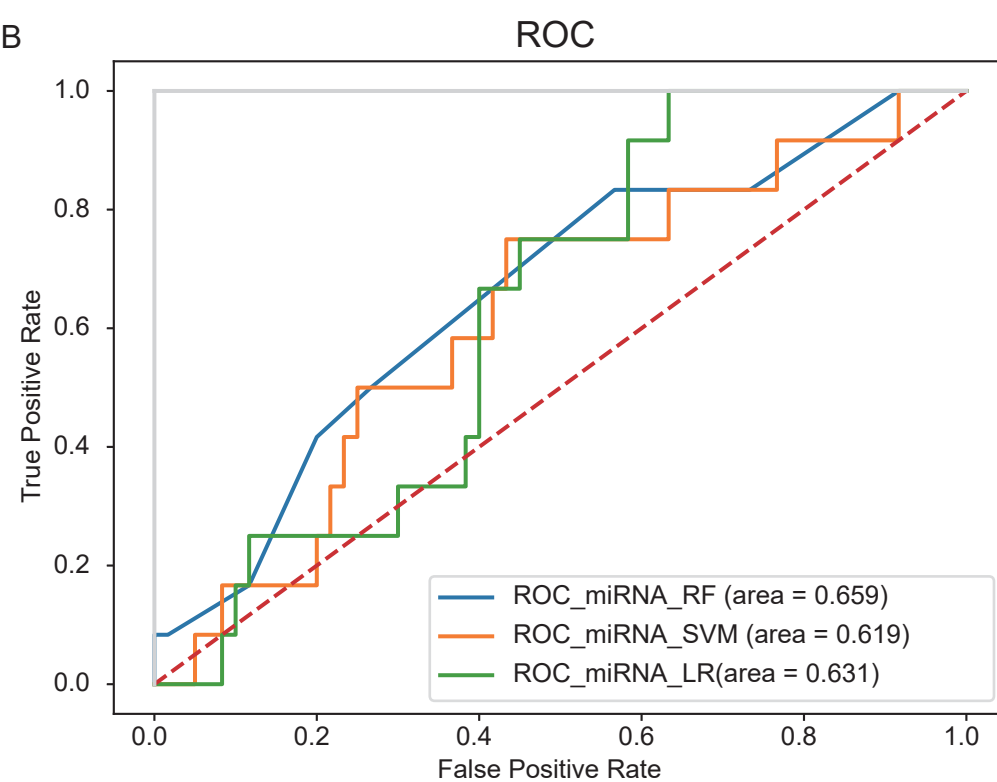

C

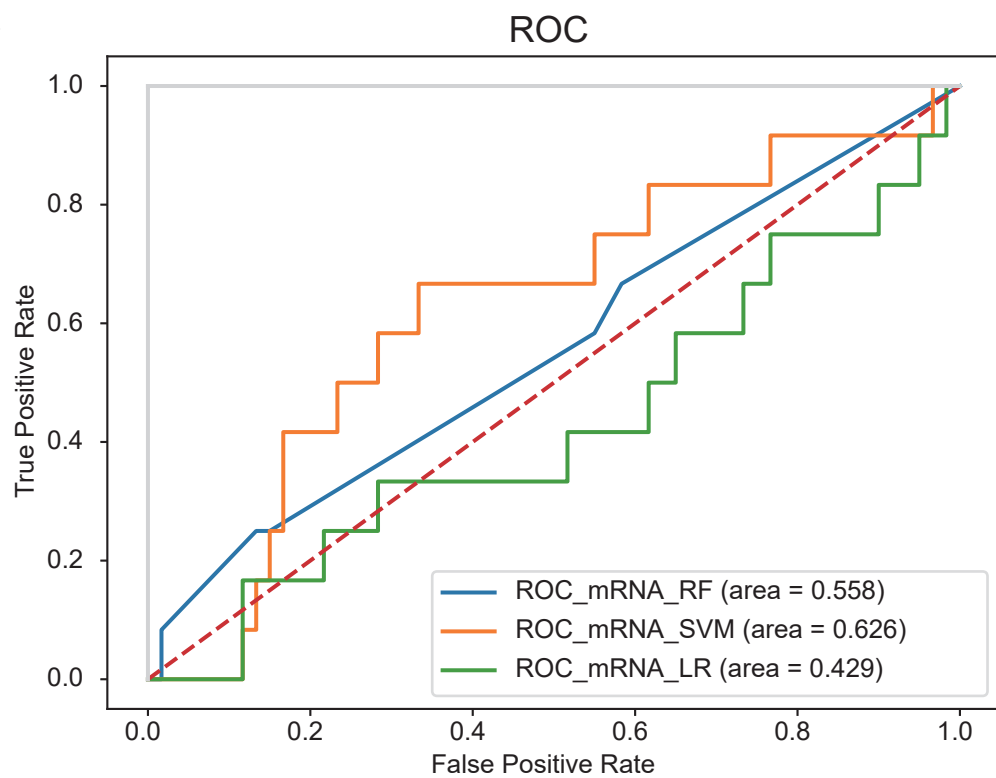

Supplement: Supplementary Figure 3 — Heatmap of the top 50 differentially expressed miRNA. [file DataSheet_3.pdf]

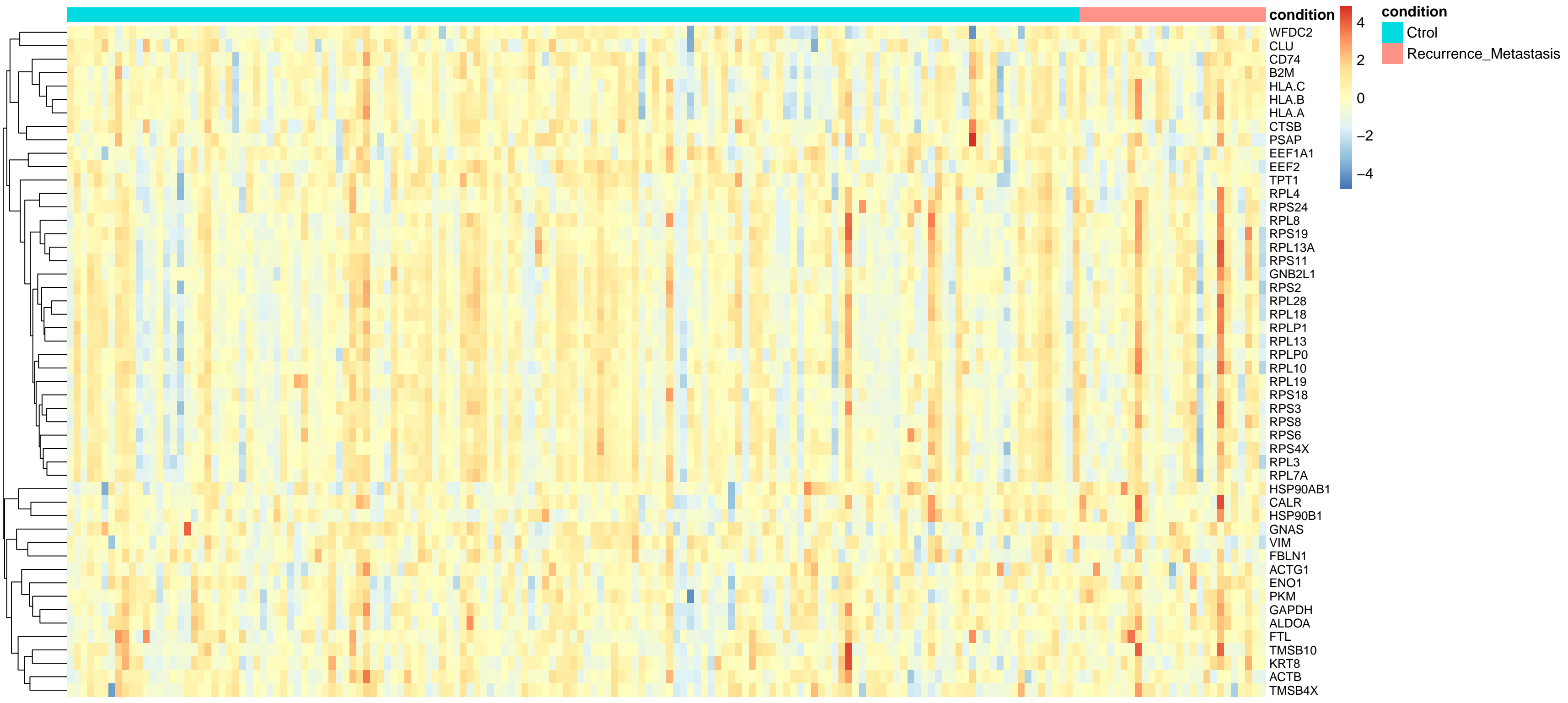

Supplement: Supplementary Figure 4 — Prediction performance of three kinds of models based on CNV data (A), miRNA data (B) and mRNA data (C). [file DataSheet_4.pdf]
